# Evaluation of Gliomas with Magnetic Resonance Fingerprinting with PET Correlation—A Comparative Study

**DOI:** 10.3390/cancers15102740

**Published:** 2023-05-12

**Authors:** Wolfgang Marik, Pedro Lima Cardoso, Elisabeth Springer, Wolfgang Bogner, Matthias Preusser, Georg Widhalm, Gilbert Hangel, Johannes A. Hainfellner, Ivo Rausch, Michael Weber, Victor Schmidbauer, Tatjana Traub-Weidinger, Siegfried Trattnig

**Affiliations:** 1 Division of Neuroradiology and Musculoskeletal Radiology, Department of Biomedical Imaging and Image-Guided Therapy, Medical University of Vienna, 1090 Vienna, Austria; wolfgang.marik@meduniwien.ac.at (W.M.);; 2 High-Field MR Centre, Department of Biomedical Imaging and Image-Guided Therapy, Medical University of Vienna, 1090 Vienna, Austria; 3 Institute of Radiology, Hietzing Hospital, 1130 Vienna, Austria; 4 Division of Oncology, Department of Internal Medicine I, Medical University of Vienna, 1090 Vienna, Austria; 5 Department of Neurosurgery, Medical University of Vienna, 1090 Vienna, Austria; 6 Division of Neuropathology and Neurochemistry, Department of Neurology, Medical University of Vienna, 1090 Vienna, Austria; 7 Division of Nuclear Medicine, Department of Biomedical Imaging and Image-Guided Therapy, Medical University of Vienna, 1090 Vienna, Austria

**Keywords:** MR fingerprinting, PET, gliomas, brain/central nervous system cancers, magnetic resonance imaging

## Abstract

**Simple Summary:**

Qualitative assessments represent the current mainstay for neuro-oncologic MRI. Additionally, PET provides important information about metabolic aspects, which is of utmost importance for treatment monitoring. While PET provides quantitative data based on tracer uptake, qualitative MRI is currently limited by subjective judgments and low inter-scanner reliability. MR fingerprinting is considered a promising approach to overcome these limitations by generating imaging data for both qualitative and quantitative assessments, based on a single sequence acquisition. This novel approach characterizes the investigated tissue by retrieving quantitative MR metrics, based on each voxels unique signal fingerprint. In this investigation, PET- and MR fingerprinting were compared to enhance the predictability of tumor-related characteristics. While both modalities revealed high predictability, the results differed, reflecting their diverse underlying mechanisms. However, the combined use of PET- and MR fingerprinting provides promising potential to retrieve tissue-specific properties, complemented by metabolic information, which, therefore, increases the value of hybrid imaging in neuro-oncology.

**Abstract:**

Objectives: Advanced MR imaging of brain tumors is still mainly based on qualitative imaging. PET imaging offers additive metabolic information, and MR fingerprinting (MRF) offers a novel approach to quantitative data acquisition. The purpose of this study was to evaluate the ability of MRF to predict tumor regions and grading in combination with PET. Methods: Seventeen patients with histologically verified infiltrating gliomas and available amino-acid PET data were enrolled. ROIs for solid tumor parts (SPo), perifocal edema (ED1), and normal-appearing white matter (NAWM) were selected on conventional MRI sequences and aligned to the MRF and PET images. The predictability of gliomas by region and grading as well as intermodal correlations were assessed. Results: For MRF, we calculated an overall predictability by region (SPo, ED1, and NAWM) for all of the MRF parameters of 76.5%, 47.1%, and 94.1%, respectively. The overall ability to distinguish low- from high-grade gliomas using MRF was 88.9% for LGG and 75% for HGG, with an accuracy of 82.4%, a ppV of 85.71%, and an npV of 80%. PET positivity was found in 13/17 patients for solid tumor parts, and in 3/17 patients for the edema region. However, there was no significant difference in region-specific MRF values between PET positive and PET negative patients. Conclusions: MRF and PET provide quantitative measurements of the tumor tissue characteristics of gliomas, with good predictability. Nonetheless, the results are dissimilar, reflecting the different underlying mechanisms of each method.

## 1. Introduction

Central nervous system (CNS) tumor classification has long been based on histological findings to define different entities of gliomas. The current WHO classification update of CNS tumors in 2021 [1] provides us with a hybrid taxonomy that incorporates numerous molecular and clinicopathologic changes representing the current nomenclature of CNS neoplasms.

Over the years, several MRI methods have been investigated for quantitative imaging features that correlate with tumor margins, histology, or genetic markers to predict outcomes or guide surgery [2]. The aim of MRI research, therefore, has been to acquire robust multiple quantitative parameters. However, conventional quantitative methods developed thus far provide information about only a single parameter at a time and are often sensitive to scanner-specific imperfections.

MR fingerprinting (MRF), a recently developed MRI technique, takes a completely different approach to data acquisition, post-processing, and visualization [3,4]. By using a pseudo-randomized and highly undersampled signal acquisition, unique temporal signal evolutions, or so-called “fingerprints”, from different tissues can be simultaneously acquired by a single fast sequence. With post-acquisition processing that involves a pattern-matching algorithm, specific quantitative maps can be acquired with tumor tissue-specific relaxation parameters, enabling further quantitative multiparametric analysis of tissue components [5]. In addition to MRI-derived features, the assessment of metabolic features, in terms of PET, has been shown to be highly valuable for the determination of biologically aggressive tumor parts and mutational status [6].

There have already been promising preliminary reports on the use of MRF in different clinical applications [4,7,8]. Haubold et al. assessed features of amino-acid PET/MRI and MRF with respect to the grading and mutational status of gliomas [9]. However, most of these studies were limited to a single entity (MRF or PET) and did not consider motion sensitivity, which has a significant impact on the generated quantitative maps [3,10].

The aim of our study was, therefore, to evaluate the predictive ability of MRF for the depiction of tumor location and grading and to compare this ability with amino-acid PET data.

## 2. Materials and Methods

This study was performed in compliance with the Declaration of Helsinki and approved by the local ethics review committee. Written, informed consent was obtained from all of the study participants prior to examination. Seventeen patients with diffuse gliomas (nine female, eight male), with a mean age of 47 years (age range, 23–77 years), were included in this study.

Tumors were histopathologically classified according to the WHO classification 2021. Fourteen of the seventeen diffuse gliomas showed an IDH-mutation, and three were IDH-wildtype. One diffuse astrocytoma CNS WHO grade 2 was partially resected six years and four months before the MRI acquisition, and one anaplastic astrocytoma CNS WHO grade 3 was biopsied six months before the MRI acquisition. For clinical and histopathological details, see Table 1.

The conventional MR brain tumor protocol comprised the sequences and parameters listed in Table 2 and was performed on a 3 T MR scanner (MAGNETOM Trio, Siemens Healthcare, Erlangen, Germany) using a head coil with 32 receive channels. The MRF protocol was performed on a 3 T MR scanner (MAGNETOM PrismaFit, Siemens Healthcare, Erlangen, Germany) using a head/neck coil with 64 receive channels.

In the IDH-wildtype cohort, the MRF protocol and the conventional tumor protocol were performed within a maximum of three days for the glioblastomas. To avoid corruption of MRF datasets, intravenous gadolinium-based contrast administration in the conventional protocol was performed either a minimum of 12 h before, or after MRF.

### 2.1. MR Fingerprinting Protocol

Motion artifacts, particularly due to through-plane motion, may cause significant deviations of the quantitative parametric values [11]. To avoid this bias, two consecutive MRF repetitions were measured, and an extra semi-automatic quality control algorithm was included via automatic calculation of “on-the-fly” difference maps between segments of the MRF signal temporal evolution and their dictionary evolution, followed by subsequent qualitative visual assessment. Distinct spatial patterns could be visualized at different strengths. These were manually labelled as either motion-corrupted or not (with severity levels of “low”, “medium”, and “strong”). Only slices classified with “no” or “low” levels of motion artifacts were used.

Within the MRF protocol, conventional MRI techniques were performed to localize the pathologic region and for co-registration purposes. For the complete MRF protocol, see Table 3.

The MRF sequence was based on a 2D fast imaging with steady-state precession (FISP) spiral readout with a prescan-based B1+ correction [12] and 1500 time points acquired at variable TR and flip angles.

The spiral readout was designed with an under-sampling factor of 48 and a spiral angle increment of 82.5°. A 256 × 256 matrix size with a field-of-view (FOV) of 256 mm was used, resulting in an in-plane resolution of 1.0 mm^2^. The scan time per slice was 20 s. The number of acquired slices was generally ten and increased if required according to the size of the tumor and its surrounding tissue. A slice thickness of 5 mm and an inter-slice gap of 1 mm were used. The Siemens auto-align scout localizer was used to attain anterior commissure (AC)–posterior commissure (PC) slice alignment.

Voxel-wise T1 and T2 parameters were derived by matching the temporal signal pattern from the acquired 1500 TRs to the entries of a pre-calculated dictionary computed by solving the Bloch equations. T1 and T2 parameter maps were calculated by prototype software integrated into the scanner’s image reconstruction pipeline. The covered parameter range of the dictionary was 10–4500 ms for T1 and 2–3000 ms for T2. The voxel-wise matching process identified the most similar dictionary entry by determining the inner product between each MRF time-course and all of the dictionary entries with maximum value.

### 2.2. PET

PET/CT imaging was performed with a Siemens Biograph 64 TruePoint system (Siemens Healthcare, Knoxville, TN, USA) using either FET(O-(2-[^18^F]-Fluorethyl)-L-Tyrosine) or MET ([^11^C]-methionine). The acquisition protocol for FET PET-CT consisted of a 20-min single-bed position PET acquisition 20 min after the injection of ~200 MBq of FET intravenously. The acquisition protocol for MET PETCT consisted of a 10 min static image acquisition 20 min post injection of ~700 MBq MET intravenously. For both tracers, a low-dose CT was acquired for attenuation correction. Using a matrix of 128 × 128, PET image reconstruction was carried out. For FET, an ordered subset expectation maximization algorithm (OSEM) of two iterations and 24 subsets and a post-reconstruction Hahn filtering with a FWHM of 5 mm was used, while for MET, an OSEM of six iterations and 21 subsets with no post-reconstruction filtering was used.

### 2.3. Co-Registration

MPRAGE images of the advanced brain tumor protocol and 3D datasets of PET-CT were iteratively co-registered to those of the MRF protocol’s MP2RAGE using the MR quantitative tool (MRQT) software prototype (v.0.3.5, Siemens Healthineers, Erlangen, Germany) and were reformatted to the same orientation via trilinear interpolation.

### 2.4. Region-of-Interest (ROI) Evaluation

**Figure 1:**
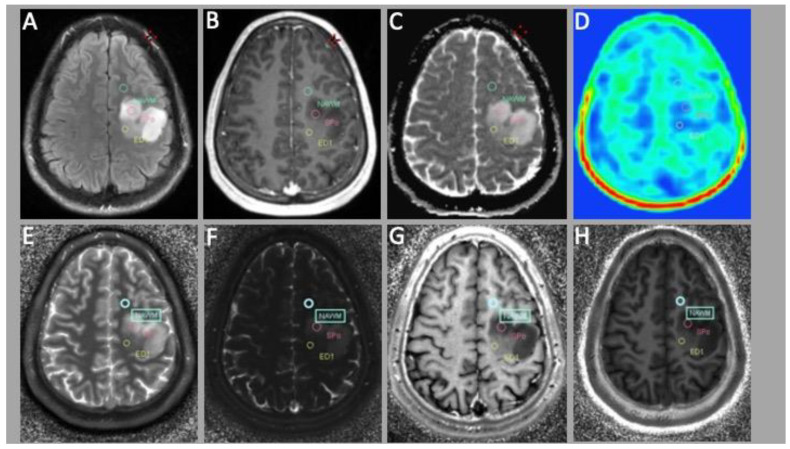
Patient with an astrocytoma Gr. 2 according to the WHO 2021 classification with a MET PET-negative tumor. From upper left to lower right: conventional MRI sequences—(**A**) FLAIR (Fluid attenuated inverse recovery); (**B**) T1w images with contrast enhancement; (**C**) ADC map; and (**D**) MET PET image. MRF images—(**E**) T1 map; (**F**) T2 map; (**G**) R2 map; and (**H**) R1 map.

**Figure 2:**
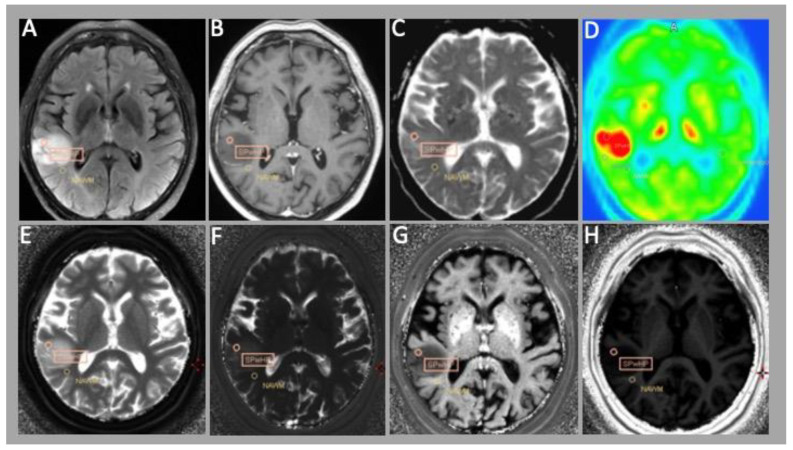
Patient with an astrocytoma Gr. 3 according to WHO 2021 classification with MET PET-positive tumor. From upper left to lower right: conventional MRI sequences—(**A**) FLAIR (Fluid attenuated inverse recovery); (**B**) T1w images with contrast enhancement; (**C**) ADC map; and (**D**) methionine PET image. MRF images—(**E**) T1 map; (**F**) T2 map; (**G**) R2 map; and (**H**) R1 map.

The following ROIs were selected on multiple slices by two experienced neuroradiologists (E.S. and W.M.), in consensus, based on the standard MRI tumor protocol using the MRQT prototype (Version 0.3.5): (i) the solid part of the tumor (SPo); (ii) perifocal edema less than or equal to 1 cm distant from the tumor (ED 1); (iii) perilesional NAWM less than or equal to 1 cm distant from the tumor or peritumoral edema (NAWM); and (iv) NAWM of the contralateral lobe (cNAWM) in normal-appearing healthy tissue to calculate a correlation coefficient for white matter (see also Figure 1 and Figure 2).

In non-enhancing tumors, the solid part of the tumor was defined as hyperintense on the FLAIR but not fluid-like with mass effect. Otherwise, the solid part with substantial contrast uptake was chosen. Edema was defined on FLAIR images as a hyperintense signal-alteration of the parenchyma without significant mass effect or architectural distortion. Areas of hemorrhage were excluded, and in cNAWM, care was taken to exclude white matter signal abnormalities. All of the selected ROIs were automatically copied to all of the other MRI sequences and PET images.

### 2.5. Statistical Analysis

All of the statistical computations were performed by an experienced statistician (M.W., >20 years of experience) using IBM SPSS Statistics for Windows version 26. All of the presented results are region-based. Metric data are described using mean and standard error. The predictability of the region and grading were assessed. Bivariate correlation between metric data was calculated using the Spearman’s Rho and Pearson correlation coefficient (r). A *p*-value ≤ 0.05 was considered significant. To avoid an error of the second type, no multiplicity corrections were performed.

## 3. Results

### 3.1. MRF

**Figure 3:**
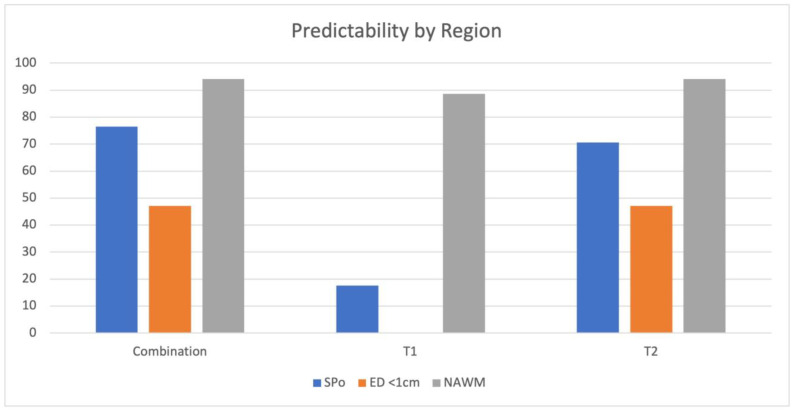
Overall predictability for all regions (SPo—solid part; ED < 1 cm—edema less than 1 cm of solid tumor parts; and NAWM—normal-appearing white matter in the same hemisphere as the tumor) given in percentage on MRF maps (T1, T2, and a combination map).

The overall predictability for the differentiation of the regions of SPo, ED1, and NAWM on the T1 maps was calculated at 17.6%, 0%, and 88.2%, respectively, and on the T2 maps at 70.6%, 47.1%, and 94.1%, respectively. By combining all of the MRF parameters, we calculated a predictability for SPo, ED1, and NAWM of 76.5%, 47.1%, and 94.1%, respectively (Figure 3).

By further subdividing into high-grade gliomas (HGG) and low-grade gliomas (LGG), we calculated a predictability for SPo, ED 1, and NAWM of all of the MRF sequences. On the T1 maps, we calculated 77.8%, 55.6%, and 55.6% for LGG, and 25%, 0%, and 0% for high-grade tumors (HGG), and on T2 maps we calculated 88.9%, 55.6%, and 44.4% for LGG, and 37.5%, 37.5%, and 50% for HGG. Considering the low overall number of patients, no multivariate prediction models were calculated for the discrimination of different regions for low- and high-grade tumors separately.

**Figure 4:**
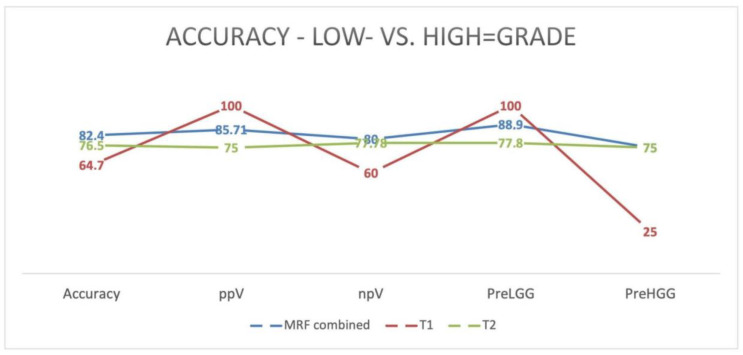
Accuracy of prediction for LGG vs. HGG given in percentages for all MRF maps (T1, T2, and combined). Abbreviations: ppV—positive predictive value; npV—negative predictive value; PreLGG—predictability for low-grade gliomas; and PreHGG—predictability for high-grade gliomas.

The overall predictability to distinguish low-(preLGG) from high-grade gliomas (preHGG) using MRF was 88.9% for LGG and 75% for HGG. The overall accuracy, therefore, was 82.4%, with a ppV (positive predictive value) of 85.71% and an npV (negative predictive value) of 80% (Figure 4).

By further subdividing this into single maps, we achieved accurate predictability on T1 maps of 100% for LGG, 25% for HGG with an accuracy of 64.7%, a ppV of 100%, and an npV of 60%; and, on T2 maps, 77.8% for LGG, 75% for HGG with an accuracy of 76.5%, a ppV of 75%, and an npV of 77.78.

### 3.2. PET Evaluation

PET-positive regions were defined by a tumor/background (T/B) ratio cut-off value of >1.4 for MET PET and >1.6 for FET PET. With these T/B ratios, we evaluated the 51 MRI-defined brain regions of the glioma patients. Overall amino acid-positive regions were seen in 13/17 patients for the SPo region and in 3/17 patients for the ED1 region, and there was no elevated amino-acid tracer uptake over the T/B ratio cut-off in 17/17 patients for the NAWM region.

The measured T1 mean values in the PET-positive regions were 1491 ms (sd 784.02 ms) for SPo and 1350.96 ms (sd 377.38 ms) for ED 1; in the PET-negative regions, the values were 1544.42 ms (sd 1091.83 ms) for SPo, 1338.02 ms (sd 190.7 ms) for ED1, and 979 ms (sd 71.12 ms) for NAWM, respectively.

**Figure 5:**
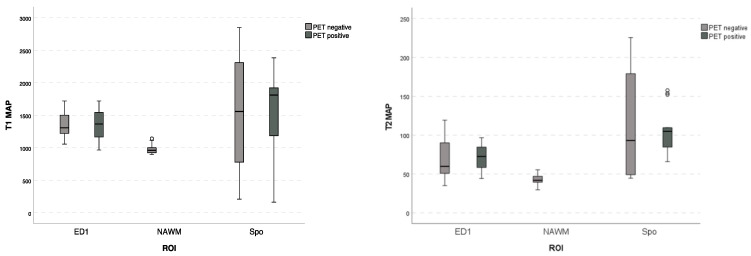
Boxplots with T1 and T2 value maps with standard deviation of each region (SPo, ED1, and NAWM) comparing PET-positive and PET-negative patients. Different mean MRF values by different regions can be seen; however, there is no significant difference in measured MRF values of the same region independent of PET status. NAWM regions of all patients were PET-negative. (SPo—solid part; ED < 1 cm—edema less than 1 cm of solid tumor parts; and NAWM—normal-appearing white matter in the same hemisphere as the tumor).

The measured T2 mean values in the PET-positive regions were 105.69 ms (sd 31.17 ms) for SPo and 71.06 ms (sd 26.18 ms) for ED 1; in the PET-negative regions, the values were 114.05 ms (sd 84.17 ms) for SPo, 67.11 ms (sd 26.67 ms) for ED1, and 42.31 ms (sd 7.3 ms) for NAWM, respectively (Figure 5).

Comparing MRI-defined solid tumor regions with PET-positive regions, we found matching results for tumor in 13/17 patients and in 4/17 patients, as well as non-matching results overall. By subdividing into LGG and HGG, we found matching results in 7/9 patients for the LGG group and in 6/8 patients for the HGG group.

### 3.3. Correlation of MRF and PET

No direct correlation between the MRF values and PET values for the regions SPo, ED 1, and NAWM was found with either Pearson’s correlation or Spearmen’s Rho rank test.

## 4. Discussion

MRF is still a rather new methodology that provides multiparametric quantitative information in a single acquisition. By using pseudo-randomized sequence parameters (e.g., TR, TE, TI, and flip angles), it is possible to acquire multiple tissue properties simultaneously, thereby providing quantitative maps. Rather recently, MRF has been shown to be of value in the characterization of gliomas and in the potential discrimination of IDH status [5,7,13]. However, due to this rather new technique, data on MRF and PET in gliomas are still scarce.

In our study, we scanned 17 patients with gliomas with MRF and amino-acid PET. In the PET examinations, either FET or MET were used in equal amounts. We started by comparing the predictive ability of MRF by region defined with conventional MRI (e.g., SPo, ED1, and NAWM). We observed a good prediction of solid tumor regions on T2 maps alone. However, the prediction of solid tumor parts further increased slightly by combining all of the MRF parameters as reported above. As expected, the generated T1 maps added only a little additional information. This is in accordance with the literature, as T2 maps are more suitable for tumor delineation, especially in low-grade gliomas, while T1 maps seem to be more useful in the delineation of edema [13,14]. However, we observed rather poor predictability of peritumoral edema in all of the sequences. This also reflects the literature as Badve et al. [7] have described the difficulty of distinguishing between solid tumor and peritumoral edema. By nature, gliomas are expected to grow along nerve cells, which causes problems in the exact delineation of these tumors on imaging. This correlates with reports of neoplastic cells being identified in peritumoral regions as far as 2.5 cm from the enhancing tumor margin without any corresponding signal abnormality on images [15]. As we had many low-grade gliomas included, which are known to have a rather small perilesional edema, this seems to be reflected in our data.

Further, we compared the predictability of MRF to distinguish between low- and high-grade tumors. We observed rather superior predictability when using all of the data generated with the MRF sequence, with an accuracy of 82% and a ppV of 85%. Interestingly, T1-generated maps achieved much higher predictability values for low-grade tumors, while in high-grade tumors, the generated T1 and T2 maps showed more even results. Therefore, rather good but not perfect results were achieved in identifying low-grade tumors, particularly while we had rather steady results in high-grade tumors.

The presented results are in contrast with Badve et al. [7], who failed to show demonstrable T1 and T2 differences for solid tumor regions of LGGs and GBMs. However, T1 and T2 changes, which are enhanced by mapping, are thought to reflect changes in cellular density, anaplasia, water content, and microvascular proliferation in GBMs compared to LGGs [7,16,17], and T1 maps are believed to best reflect differences in water content. This might explain our good differentiation results for LGG and HGG using T1 maps as it might reflect less cellular density to a greater degree, close to that of normal water tissue content of LGG compared to HGG.

Comparing these MRI ROI-defined results with amino-acid PET data, 13/17 PET positive tumors were observed in the SPo region. Interestingly, the four PET-negative results were seen in both the HGG (2/8) and in the LGG (2/9) group. Nonetheless, according to literature [18,19], the majority of HGG seems to be PET-positive, which was also seen in this small study population. Looking more closely into the database, both PET-negative brain tumors in the HGG group turned out to be diffuse astrocytomas formerly classified as grade 2 and 3 in 2016. However, the grade 2 tumor was an IDH-wildtype and consequently regraded as HGG based on the 2021 WHO CNS classification [1]. Although IDH mutation status has been established for the prognostic estimation of gliomas, amino-acid metabolism as imaged by MET or FET is also thought to mirror prognostic information [20,21]. Therefore, we assume that this reflects the different metabolic rates in these tumors. Nevertheless, we found 3/17 PET-positive results for the ED1 regions of HGGs, which has been confirmed by others [22] who demonstrated hypermetabolism and impaired CVR (cerebrovascular reactivity) beyond the standard MRI-defined tumor border, suggestive of active tumor infiltration. Nonetheless, comparing the MRF values by regions (SPo, ED1, and NAWM), we found different interregional values but failed to show significant differences by comparing PET-positive and PET-negative values of the same region, probably because of the small number of investigated gliomas. Another explanation for our discordant results might be that, while PET measurements reflect the current metabolic amino-acid status of the tumor, MRF measured values tend to show the particular physical status of tissue at a certain time point. Both methods are altered by tumoral changes but mirror different underlying pathomechanisms. This is also supported by others, which emphasizes the importance of a comprehensive multimodal approach and highlights the value of both methods [9,19,23,24].

Limitations of this study include the use of two 3T MR scanners (the advanced brain tumor protocol and the MRF protocol), although from a single manufacturer. Moreover, it has been shown that MRF results are stable across different 3 T MR hardware setups of the same manufacturer [10].

Furthermore, our PET data were acquired on a single PET/CT scanner, which might have caused a certain alignment error. However, we tried to overcome this issue by using special software that was designed to align the acquired 3D datasets as described above.

Another limitation of this study is the limited number of enrolled patients; however, the presented data are preliminary, and larger patient cohorts are needed in future trials.

## 5. Conclusions

We conclude that MRF and PET provide quantitative measurements with good predictability of MRF on the grading and tumor regions of gliomas. Nonetheless, the results are dissimilar, reflecting the different underlying mechanisms of each method.

However, the use of a combined PET/MR scanner with an application for MRF could offer the possibility to replace currently used advanced brain tumor protocols in the future with a promising new approach for neuroimaging by providing information about not only tissue type but also about the current metabolic status of brain tumors in a short, quantitative, and reproducible way.

## Figures and Tables

**Table 1:** Patient characteristics—age, sex, histology, grading, and IDH/MGMT/1p/19q status (WHO 2021 and 2016). PET tracers (FET—F-18-Fluoroethyl L-tyrosine, MET—methionine) for each patient.

Nr	Age	PET	Gender	Classification (WHO 2021)	Classification (WHO 2016)	MGMT-Status (Methyliert = 1 Unmethyliert = 0)	1p/19q (1 = Codel, O = n.a.)	IDH (Mutant = 1 Wildtyp = 0)
1	23	MET	m	Astrocytoma, IDH-mutant (CNS WHO grade 2)	Diffuse astrocytoma, IDH-mutant (WHO Gr. II)	1	0	1
2	58	MET	m	Astrocytoma, IDH-mutant (CNS WHO grade 3)	Anaplastic astrocytoma, IDH-mutant (WHO Gr. III)	0	0	1
3	46	MET	f	Astrocytoma, IDH-mutant (CNS WHO grade 4)	Glioblastoma, IDH-mutant (WHO Gr. IV)	1	0	1
4	52	FET	m	Glioblastoma, IDH-wildtype (CNS WHO grade 4)	Glioblastoma, IDH-wildtype (WHO Gr. IV)	1	0	0
5	29	MET	m	Astrocytoma, IDH-mutant (CNS WHO grade 3)	Anaplastic astrocytoma, IDH-mutant (WHO Gr. III)	1	0	1
6	33	FET	m	Astrocytoma, IDH-mutant (CNS WHO grade 2)	Diffuse astrocytoma, IDH-mutant (WHO Gr. II)	1	0	1
7	54	MET	f	Astrocytoma, IDH-mutant (CNS WHO grade 2)	Diffuse astrocytoma, IDH-mutant (WHO Gr. II)	0	0	1
8	77	FET	f	Astrocytoma, IDH-mutant (CNS WHO grade 2)	Diffuse astrocytoma, IDH-mutant (WHO Gr. II)	1	0	1
9	46	MET	f	Astrocytoma, IDH-mutant (CNS WHO grade 2)	Diffuse astrocytoma, IDH-mutant (WHO Gr. II)	1	0	1
10	52	FET	m	Oligodendroglioma, IDH-mutant and 1p/19q codeleted (CNS WHO grade 2)	Oligoendroglioma, IDH-mutant and 1p/19q codeleted (WHO Gr. II)	1	1	1
11	57	FET	m	Astrocytoma, IDH-mutant (CNS WHO grade 2)	Diffuse astrocytoma, IDH-mutant (WHO Gr. II)	0	0	1
12	65	MET	f	Glioblastoma, IDH-wildtype (CNS WHO grade 4)	Anaplastic astrocytoma, IDH-wildtype (WHO Gr. III)	0	0	0
13	51	FET	m	Oligodendroglioma, IDH-mutant and 1p/19q codeleted (CNS WHO grade 3)	Anaplastic oligodendroglioma, IDH-mutant and 1p/19q codeleted (WHO Gr. III)	1	1	1
14	27	FET	m	Glioblastoma, IDH-wildtype (CNS WHO grade 4)	Diffuse astrocytoma, IDH-wildtype (WHO Gr. II)	0	0	0
15	28	MET	f	Astrocytoma, IDH-mutant (CNS WHO grade 3)	Anaplastic astrocytoma, IDH-mutant (WHO Gr. III)	1	0	1
16	39	FET	f	Oligodendroglioma, IDH-mutant and 1p/19q codeleted (CNS WHO grade 2)	Oligodendroglioma, IDH-mutant and 1p/19q codeleted (WHO Gr. II)	1	1	1
17	61	FET	m	Oligodendroglioma, IDH-mutant and 1p/19q codeleted (CNS WHO grade 2)	Oligodendroglioma, IDH-mutant and 1p/19q codeleted (WHO Gr. II)	1	1	1

Abbreviations: PET—positron emission tomography; n.a.—not accessible; IDH—isocitrate dehydrogenase; and MGMT—O^6^-methylguanine-DNA-methyltransferase.

**Table 2:** Conventional tumor protocol used for 3 T.

	2D ax T2 FLAIR	2D T2 ax	DWI ax	3D SWI ax	3D T1 ax pre	2D T2 cor	PWI ax	3D T1 ax post	2D CSI	DTI ax
	TSE + IR	TSE	EPI-SE	GRE	MPRAGE	TSE	SS-EPI	MPRAGE	S-LASER	EPI-SE
Voxel dimensions	0.9 × 0.9	0.8 × 0.6	1.8 × 1.8	0.9 × 0.9	1 × 1	0.4 × 0.4	1.8 × 1.8	1 × 1	10 × 10	2 × 2
Matrix size	256 × 256	250 × 384	128 × 128	256 × 192	256 × 256	531 × 640	128 × 128	256 × 256	16 × 16	128 × 128
No. slices	36	40	30	80	192	56	19	192	1	65
Field of view (mm^2^)	230	210	230	230	220	230	230	220	160	256
Slice thickness, mm	4	3	5	1.75	1	3	5	1	10	2
TE (ms)	100	88	78	20	3.79	115	32	3.79	135	83
TI (ms)	2500	-	-	-	1100	-	-	-	-	-
TR (ms)	9220	3490	4000	28	1800	4290	1400	1800	1510	8000
TA (min:s)	4:38	1:25	1:38	3:52	5:44	3:40	1:17	5:44	6:07	4:38
GRAPPA factor	-	-	2	2	-	2	2	-	-	2
RB/pixel, Hz/pixel	170	199	1502	120	200	176	1346	200	1200	1562
FA (°)	150	120	-	15	12	120	90	12	90	-
Fat saturation	yes	no	yes	No	no	No	yes	no	yes	yes

2D T2 FLAIR TSE, 2D T2-weighted fluid-attenuated inversion recovery turbo spin echo; 2D T2 TSE, 2D T2-weighted turbo spin echo; DWI, diffusion-weighted echo-planar spin echo imaging; 3D SWI, 3D susceptibility-weighted gradient recalled echo imaging; 3D T1 MPRAGE (pre-/post), 3D magnetization prepared rapid acquisition gradient echo sequence pre- and post-contrast injection; post-contrast images were collected with parameters equivalent to those of pre-contrast images; PWI, perfusion-weighted imaging with single-shot echo planar imaging; CSI, chemical shift imaging = multi voxel spectroscopy with semi-localization by adiabatic selective refocusing; and DTI, diffusion tensor echo-planar spin echo imaging.

**Table 3:** MRF protocol used for 3 T.

	2D ax T2 FLAIR	3D T1 Sag	Multi-Echo Spin Echo	MRF
	TSE + IR	MP2RAGE	(T2 Map)	
Voxel dimensions (mm^2^)	0.6 × 0.6	1.0 × 1.0	0.7 × 0.7	1.0 × 1.0
Matrix size	384 × 276	256 × 216	320 × 257	256 × 256
No. slices	10	160	10	10–13
Field of view (mm^2^)	230 × 166	256 × 216	230 × 180	256 × 256
Slice thickness (mm)	5.0	1.0	5.0	5.0
TE (ms)	126	2.98	12.6, 25.2,... 201.6	2
TI (ms)	2500	700, 2500	–	21
TR (ms)	8500	5000	2100	12.1–15.0 (varied by sequence)
TA (min:sec)	3:43	8:02	3:38	3:51–4:51
Acceleration factor	1 (turbo factor: 19)	2	3	24 (inner k-space), 48 (outer k-space)
RB/pixel (Hz/pixel)	140	240	150	RX-Bandwidth: 400 kHz
FA (°)	180	4, 5	180	0–74 (varied by sequence)
Fat saturation	yes	no	no	no

FLAIR fluid-attenuated inversion recovery; TSE, turbo spin echo; MP2RAGE, magnetization prepared 2 rapid acquisition gradient echoes; and MRF, magnetic resonance fingerprinting.

## Data Availability

All of the data are available on request to the reviewers.

## Abbreviations

ADC	Apparent diffusion coefficient
CNS	Central nervous system
CVR	Cerebrovascular reactivity
ED1	Peritumoral edema
FET	Fluorethyl-L-Tyrosine MET ([^11^C]-methionine)
FLAIR	Fluid attenuated inversion recovery
FWHM	Full width at half maximum
GBM	Glioblastoma
HGG	High grade glioma
IDH	Isocitratdehydrogenase
LGG	Low-grade glioma
MET	Methionine
MGMT	Methylguanine-DNA-methyltransferase
MPRAGE	Magnetization prepared—rapid gradient echo
MRI	Magnetic resonance imaging
MRF	Magnetic resonance finger printing
NAWM	Normal appearing white matter
npV	Negative predictive value
OSEM	Ordered subset expectation maximization algorithm
PET	Positron emission tomography
ppV	Positive predictive value
ROI	Region of interest
Sd	Standard deviation
SPo	Solid tumor part
TE	Echo time
TI	Inversion time
TR	Repetition time
